# Oral plasmablastic lymphoma: A case report

**DOI:** 10.1097/MD.0000000000022335

**Published:** 2020-09-25

**Authors:** Maurizio Zizzo, Magda Zanelli, Roberta Martiniani, Francesca Sanguedolce, Loredana De Marco, Giovanni Martino, Paola Parente, Valerio Annessi, Lorenzo Manzini, Stefano Ascani

**Affiliations:** a Surgical Oncology Unit, Azienda Unità Sanitaria Locale-IRCCS di Reggio Emilia; b Clinical and Experimental Medicine PhD Program, University of Modena and Reggio Emilia, Modena; c Pathology Unit, Azienda Unità Sanitaria Locale-IRCCS di Reggio Emilia, Reggio Emilia; d Onco-Hematology Unit, Azienda Ospedaliera Santa Maria di Terni, University of Perugia, Terni; e Pathology Unit, Azienda Ospedaliero-Universitaria, Ospedali Riuniti di Foggia, Foggia; f Hematology Unit, CREO, Azienda Ospedaliera di Perugia, University of Perugia, Perugia; g Pathology Unit, Fondazione IRCCS Casa Sollievo della Sofferenza, San Giovanni Rotondo, Foggia; h Pathology Unit, Azienda Ospedaliera Santa Maria di Terni, University of Perugia, Terni, Italy.

**Keywords:** B-cells, Epstein-Barr virus, human immunodeficiency viruses, oral cavity, plasmablastic lymphoma

## Abstract

**Introduction:**

Plasmablastic lymphoma (PBL) is an uncommon and aggressive large B-cell lymphoma commonly diagnosed in human immunodeficiency viruses -positive patients. Oral cavity is the most commonly PBL affected site. Most oral PBLs presented as asymptomatic swellings, frequently associated with ulcerations and bleeding. Most cases lacked B-symptoms, suggesting a more local involvement of the disease. No standard treatment is yet for oral PBL. Five-year survival rate recorded no more than 33.5%.

**Patient concerns:**

A 39-year-old male presented to Dental Clinic with 1 month swelling of the oral cavity, in absence of any other symptoms or signs. He followed antibiotic therapy just on suspicion of an oral abscess and later oral surgical treatment on suspicion of bone neoplasm.

**Diagnosis:**

Surgical specimen analysis highlighted a diffuse infiltrate of large-sized atypical cells with plasmablastic appearance and plasma cell phenotype. Oral cavity PBL was diagnosed. Blood tests recorded mild lymphopenia and positive human immunodeficiency viruses serology.

**Interventions:**

Patient underwent chemotherapy including intrathecal methotrexate prophylaxis, in addition to a highly active antiretroviral therapy.

**Outcomes:**

At 12 months from diagnosis, patient recorded complete hematological remission.

**Conclusions:**

Oral PBL diagnosis requires a high level of suspicion and awareness both by physicians and pathologists. They should be aware of the extent of such disease which is often mistaken as oral abscess or infected tooth, thus leading to delay the most appropriate diagnostic evaluation. As PBL is an aggressive non-Hodgkin lymphoma, a delayed diagnosis might negatively impact on both treatment and survival.

## 1 Introduction

Plasmablastic lymphoma (PBL) is an uncommon and aggressive large B-cell lymphoma showing an immunoblastic and plasmablastic morphology with plasmacytic immunophenotype.^[1]^ PBL is commonly diagnosed in human immunodeficiency viruses (HIV)-positive patients, but it can also be detected in HIV-negative patients and those affected by immunosuppressive conditions.^[1–3]^ PBL etiology is unclear, although the role of Epstein-Barr virus (EBV) is frequently assumed, as it is detected in 78% cases.^[1–2]^ Moreover, due to its low incidence, PBL prognostic features are rarely understood, especially when PBL affects oral and maxillofacial regions, thus impairing an appropriate therapeutic management.^[1]^

Our case corroborates the paramount role that early clinical suspicion and correct diagnosis play in choosing the most appropriate treatment. Our patient underwent antibiotic therapy just on suspicion of an oral abscess. Later, on suspicion of bone neoplasm, a complete surgical removal of his lesion was carried out.

## 2 Case Presentation

**Figure 1:**
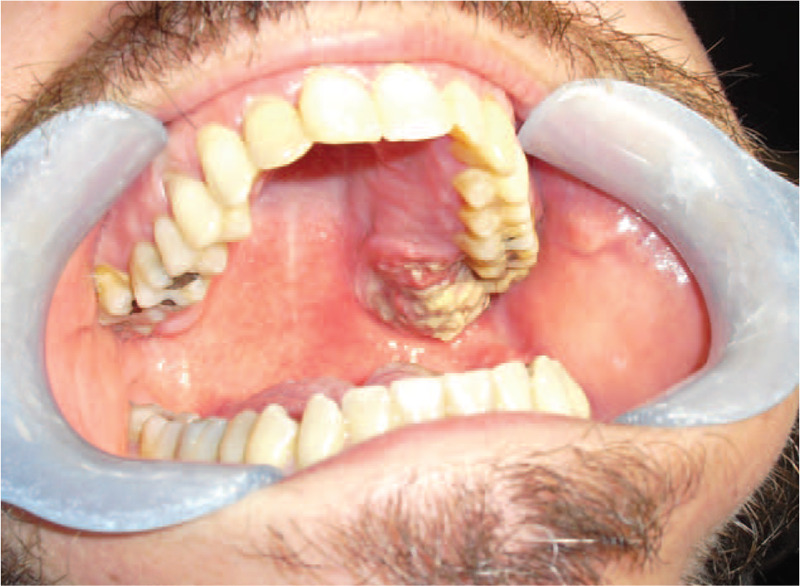
Clinical image at diagnosis highlights the lesion of the oral cavity.

**Figure 2:**
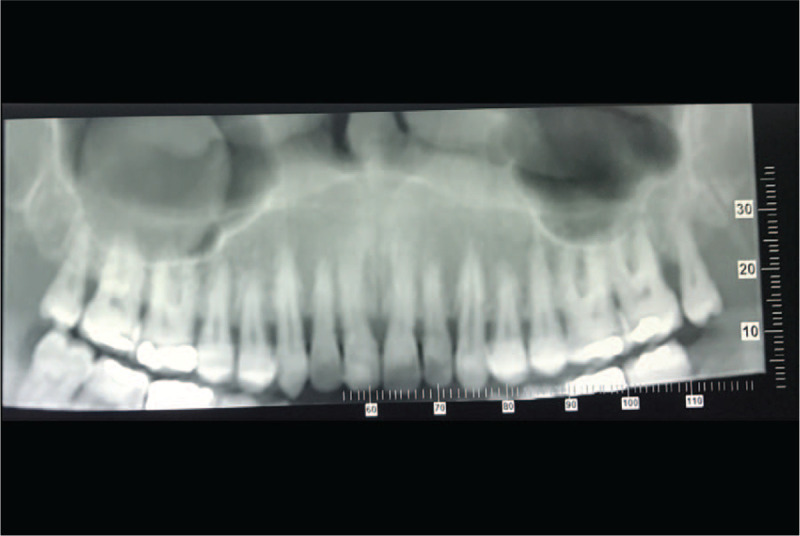
A dental panoramic radiography revealed a lesion of the upper left dental arch.

**Figure 3:**
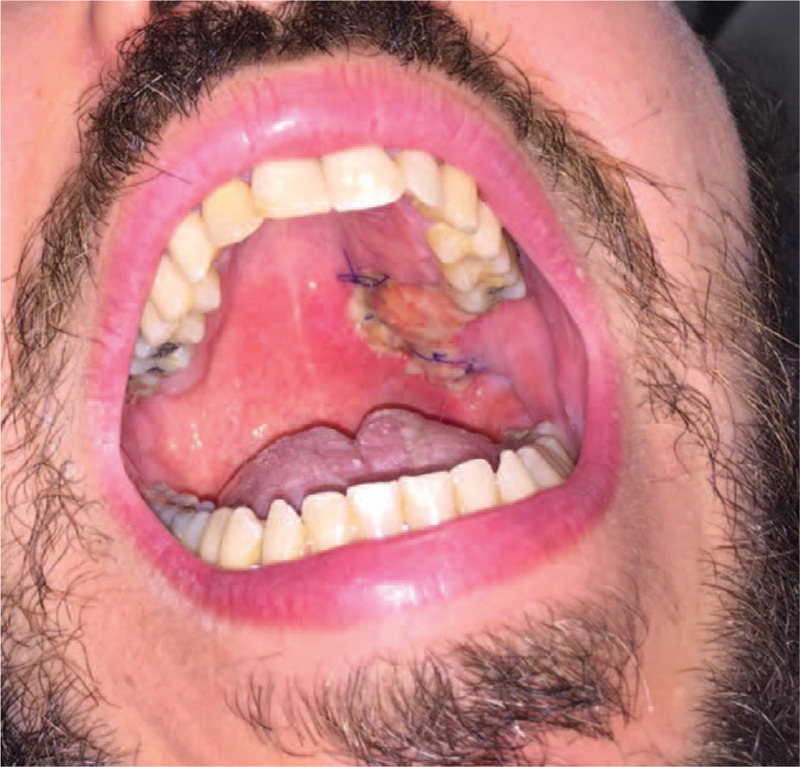
Clinical image at follow-up highlights the outcome of oral surgical treatment.

**Figure 4:**
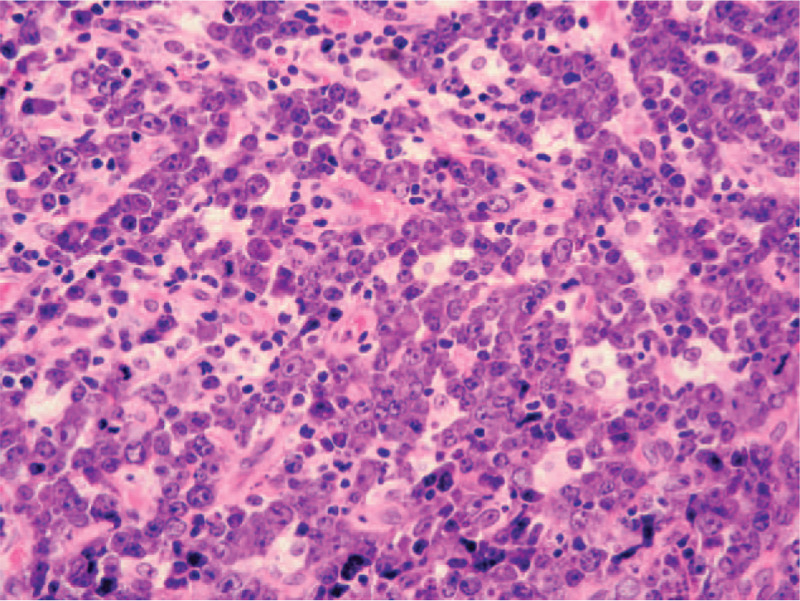
Histology highlights a diffuse infiltrate of large-sized atypical cells with a plasmablastic appearance.

**Figure 5:**
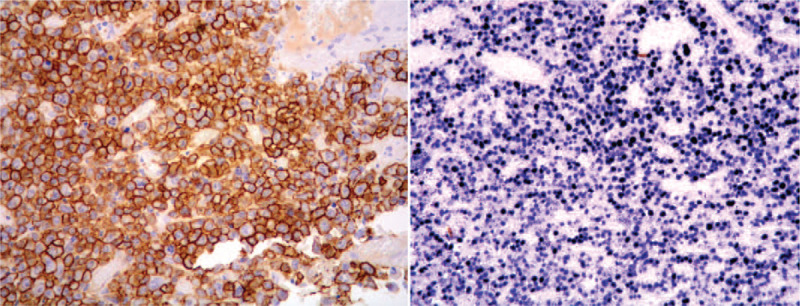
*Left* The neoplastic cells expressed a plasma cell phenotype, being positive for CD138. *Right* In situ hybridization for Epstein-Barr virus (EBV)-encoded small RNA (EBER) yielded a positive result in the neoplastic cells.

A 39-year-old male presented to Dental Clinic with 1 month swelling of the oral cavity. Following administration of oral antibiotics due to suspected oral abscess, the patient worsened (Fig. 1). His clinical records included juvenile adenoidectomy, cigarette smoking and recurrent varicella-zoster virus infections occurred in the previous year. Panoramic dental x-rays showed a lesion of left upper dental arch (Fig. 2). On suspicion of primitive bone tumor, radical excision was carried out, including the removal of a 40 mm x 18 mm soft tissue area, in addition to removal of 15 mm x 8 mm x 7 mm maxillary bone segment and 2 teeth (Fig. 3). Histology highlighted a diffuse infiltrate of large-sized atypical cells with plasmablastic appearance (Fig. 4). Neoplastic cells expressed a plasma cell phenotype which was CD138 (Fig. 5, *left*) and IRF4/MUM1 positive while being CD45, CD20 and PAX5 negative. A high proliferative index was recorded. In situ hybridization for EBV encoded small RNA showed positive results in neoplastic cells (Fig. 5, *right*). Oral cavity PBL was diagnosed. Subsequent blood tests recorded mild lymphopenia and positive HIV serology, revealing a previously unknown HIV infection.

Patient was referred to a health facility specialized in lymphoma associated with immunodeficiency, where he underwent intensive chemotherapy including intrathecal methotrexate prophylaxis, in addition to a highly active antiretroviral therapy (HAART). At 12 months from diagnosis, patient recorded complete hematological remission and he is at present in good clinical condition.

## 3 Discussion

Following Kaposi's sarcoma, non-Hodgkin lymphoma (NHL) is the most common HIV-related neoplasm.^[3]^ Being approximately 2% of all primary extranodal lymphomas, many cases affect extranodal sites, including oral cavity and jaw bones.^[1]^ During pre-HAART era, NHL incidence was 60–200 times higher in HIV-infected patients than in HIV-non-infected ones.^[2]^ However, since mid-1990 s, when HAART was first introduced, NHL incidence has seemingly been declining.^[1–3]^ Currently, NHL risk is 80 to 100 times greater in HIV-positive patients than in HIV-negative ones.^[3]^ Acquired immune deficiency syndrome related NHLs are mainly aggressive high-grade B-cell lymphomas: among them, Burkitt lymphoma, diffuse large B-cell lymphoma, primary effusion lymphoma, being PBL the most common type.^[3]^

In HIV-infected patients, PBL and NHL mainly happen at a young age, recording male predominance (5.7:1).^[1,2,4]^ PBL median age is 39 years (range 7–86).^[2]^ On the contrary, a review including more than 400 diffuse large B-cell lymphoma HIV-non-infected patients recorded a 64-year median age (range 14–98 years).^[2]^

Although being relatively rare, oral cavity is the most commonly PBL affected site, followed by gastrointestinal tract.^[1]^ Lymph nodes, skin, bone and genitourinary tract are less frequently affected.^[1]^ As regards oral cavity, gingiva is the most commonly affected area, followed by palate, where PBL usually appears as a soft tissue lesion.^[1,5]^ Most oral PBLs presented as asymptomatic swellings, frequently associated with ulcerations and bleeding. Most cases lacked B-symptoms, suggesting a more local involvement of the disease.^[1,2]^

A statistically significant association between HIV-positive patients and EBV-positive PBL ones emerged, thus suggesting that HIV infection might represent a permissive environment for chronic EBV infection and allow subsequent latency that would lead EBV-infected B-cells to malignancy.^[1–3]^

Differential diagnosis for an expanding oral lesion includes primarily infectious diseases (viridans and other streptococci, *Peptostreptococcus* spp, *Bacteroides* spp, *Actinomyces israelii*, and *Actinobacillus actinomycetemcomitans*) in addition to malignant processes as primary squamous cell tumour, metastatic tumour, Kaposi's sarcoma, and other forms of lymphoma that may occur in the oral cavity (DLBCL not otherwise specified, ALK-positive large B-cell lymphoma, primary effusion lymphoma, plasmacytomas, Burkitt's lymphoma, and multiple myeloma).^[1–3,5]^ Neoplastic cells express plasma cell markers such as CD38, MUM1, CD138, VS38c, while showing negativity for typical B-cell antigens (eg, CD20, CD79α).^[2]^

No standard treatment is yet for oral PBL.^[1–3]^ Different chemotherapy regimens have led to different results. HIV-infected patients who had been treated with HAART and chemotherapy showed better survival rates.^[1,2]^ However, many patients died in a very short follow-up time.^[1,2]^ Five-year survival rate recorded no more than 33.5%.^[2]^ EBV, B-symptoms and chemotherapy alone (without HAART) may contribute to such poor prognosis.^[1]^

## 4 Conclusion

Oral PBL diagnosis requires a high level of suspicion and awareness both by physicians and pathologists. In particular, infectious disease clinicians, dentists, stomatologists, oral and maxillofacial surgeons should be aware of the extent of such disease which is often mistaken as oral abscess or infected tooth, thus leading to delay the most appropriate diagnostic evaluation. As PBL is an aggressive NHL, a delayed diagnosis might negatively impact on both treatment and survival.

## Acknowledgments

We thank Dr Daniela Masi (AUSL-IRCCS di Reggio Emilia) for English editing.

## Author contributions

**Conceptualization:** Maurizio Zizzo, Magda Zanelli, Stefano Ascani.

**Data curation:** Maurizio Zizzo, Magda Zanelli, Roberta Martiniani, Francesca Sanguedolce, Loredana De Marco, Giovanni Martino, Paola Parente, Valerio Annessi, Lorenzo Manzini, Stefano Ascani.

**Formal analysis:** Maurizio Zizzo, Magda Zanelli, Roberta Martiniani, Stefano Ascani.

**Investigation:** Roberta Martiniani, Giovanni Martino, Stefano Ascani.

**Methodology:** Maurizio Zizzo, Magda Zanelli, Roberta Martiniani, Giovanni Martino, Stefano Ascani.

**Project administration:** Maurizio Zizzo, Magda Zanelli, Stefano Ascani.

**Resources:** Roberta Martiniani, Giovanni Martino, Stefano Ascani.

**Supervision:** Maurizio Zizzo, Magda Zanelli, Stefano Ascani.

**Writing – original draft:** Maurizio Zizzo.

**Writing – review & editing:** Maurizio Zizzo, Magda Zanelli.
